# Exploring lactylation and cancer biology: insights from pathogenesis to clinical applications

**DOI:** 10.3389/fcell.2025.1598232

**Published:** 2025-06-13

**Authors:** Zixuan Gou, Qianchuang Sun, Jiannan Li

**Affiliations:** ^1^ Department of Hematology, The Second Hospital of Jilin University, Changchun, China; ^2^ Department of Operating Theater and Anesthesiology, The Second Hospital of Jilin University, Changchun, China; ^3^ Department of General Surgery, The Second Hospital of Jilin University, Changchun, China

**Keywords:** lactylation, post-translational modification, tumor progression, prognostic biomarkers, therapeutic target

## Abstract

As a byproduct of glycolysis, lactate functions as a signaling molecule, a substrate for energy metabolism, and a regulator of the tumor microenvironment (TME). It is involved in various biological processes, including energy shuttling, tumor growth and invasion, drug resistance, and immune evasion. Lactylation, a recently identified post-translational modification (PTM), acts as a bridge between gene regulation and cellular metabolism, thus playing a crucial role in tumor biology. Similar to other epigenetic modifications, lactylation influences the spatial conformation of chromatin, modulates DNA accessibility, and regulates gene expression. It intricately participates in TME-related processes by orchestrating immune state transitions and enhancing the malignant characteristics of tumors. This review summarizes lactylation-related genes in tumors, the role of lactylation in the TME, the interactions of the genes with other metabolic pathways, and the potential mechanisms underlying tumor progression as well as their clinical implications. Despite its nascent stage, research on the epigenetic regulation of tumor-related genes by lactylation holds promise. In this review, we highlighted unresolved challenges in this field and provided insights that may guide the development of novel targeted therapies for cancer.

## 1 Introduction

Glycolysis is a pathway of glucose breakdown that enables cells to generate energy under both normoxic and hypoxic conditions. In the tumor microenvironment (TME), glycolysis is remarkably active in cancer cells, even under high-oxygen concentrations. In the 1920s, Warburg et al. identified the characteristic “aerobic glycolysis” in tumors, now known as the Warburg effect (Warburg et al., 1927). Compared with surrounding tissues, tumors consume larger amounts of glucose. Through glycolysis, tumor cells generate adenosine triphosphate (ATP) and produce excessive lactate intracellularly, even in the presence of sufficient oxygen. Lactate, an oxidizable substrate, subsequently generates a substantial amount of ATP to promote cancer cell proliferation. Despite its lower efficiency than mitochondrial oxidative phosphorylation (OXPHOS), glycolysis-dependent ATP production allows tumor cells to obtain abundant metabolic intermediates. These intermediates are essential for synthesizing lipids, nucleotides, and other biomolecules required for tumor growth. In addition to energy production, glycolysis-derived lactate drives acidification of the extracellular matrix, impairing immune cell function (Li et al., 2022) and promoting tumor metastasis and invasion (Jiang et al., 2020). Lactate is transported between cells via monocarboxylate transporters (MCTs), particularly MCT1 (Brooks, 2018; Carneiro et al., 2017), or acts as a signaling molecule through specific receptors (Roland et al., 2014).

In 2019, Zhang et al. discovered that lactate could regulate gene expression by inducing histone lactylation, a process now termed lactylation (Zhang et al., 2019). Lactylation is a lactate-mediated post-translational modification (PTM) similar to phosphorylation, acetylation, methylation, and ubiquitination. Lactate-driven epigenetic alterations represent a critical link through which metabolic states influence gene expression. Increased levels of histone and non-histone lactylation have been observed in various malignancies and are closely associated with epigenetic dysregulation and metabolic dysfunction. These processes collectively regulate intracellular signaling, gene expression, and protein functionality (Chen H. et al., 2024; Zong et al., 2024; Chen et al., 2024b). Despite its significance, the mechanisms and functions of lactylation remain unclear. An in-depth understanding of the role of this novel epigenetic modification in tumor progression may provide insights into the molecular mechanisms underlying metabolic reprogramming in cancer and facilitate the development of therapeutic strategies targeting metabolism. This review provides a comprehensive overview of the research progress of lactylation in tumors.

## 2 Histone and non-histone lactylation in the TME

The TME comprises tumor cells, cancer-associated fibroblasts (CAFs), and tumor-associated immune cells, some of which are regulated by lactate (Han et al., 2023).

Both normoxic and hypoxic cellular populations are found in the TME. Hypoxic cells, inherently lacking the ability to oxidize lactate, are typically located in regions with the highest lactate levels. These cells rely on glucose for Warburg-like metabolism, generating a large amount of lactate, which is subsequently transported to adjacent oxygenated cells via MCT1. A metabolic symbiosis has been observed between normoxic and hypoxic cells, wherein some cells undergo Warburg-like metabolism, whereas others metabolize lactate through OXPHOS-dependent pathways (Hui et al., 2017; Faubert et al., 2017). This lactate shuttling facilitates the redistribution and efficient utilization of energy substrates, which are used by tumor cells to optimize energy production and meet the demands of rapid proliferation. This division of labor in energy metabolism serves as a crucial factor contributing to the formation of a TME (Brooks, 2018; Brooks, 2020). Over the past 5 years, studies have suggested that lactylation plays an essential role in shaping many pro-tumor characteristics of cells within the TME. In addition, lactylation serves as an important epigenetic mechanism in the TME (Jiao et al., 2024; Xiong et al., 2022). High lactate levels suppress immune cells and contribute to lactylation (Apostolova and Pearce, 2022; Elia et al., 2022) on both histone and non-histone proteins. This section systematically discusses the roles of histone and non-histone lactylation in the TME, providing a valuable reference for investigating how the TME is shaped by epigenetics.

### 2.1 Histone lactylation

Histones are proteins that form the core of nucleosomes wrapped by DNA, serving as the primary structural proteins of chromosomes. PTM of histones can regulate DNA replication, transcription, and repair by altering nucleosome interactions or recruiting non-histone proteins, which are essential for maintaining homeostasis (Millan-Zambrano et al., 2022). With the advancement of high-sensitivity mass spectrometry techniques in epigenetic research, the diversity of histone PTMs continues to expand (Sabari et al., 2017). Lysine acylation of histones is a common type of PTM, with hydrophobicity, charge, and hydrocarbon chain length being different across various acylation types (Hirschey and Zhao, 2015). This modification is regulated by specific enzymes that act as “writers” and “erasers”. For instance, histone acetyltransferase (HAT) p300 is a key “writer” of histone acetylation (Moreno-Yruela et al., 2022a), whereas histone deacetylases (HDACs) remove acetyl groups from the lysine residues of both histone and non-histone proteins. Effector proteins, termed “readers,” specifically interpret modified gene information and regulate downstream signaling pathways, contributing to the dynamic regulation of lysine acylation (Moreno-Yruela et al., 2022b; Rothbart et al., 2015).

**FIGURE 1:**
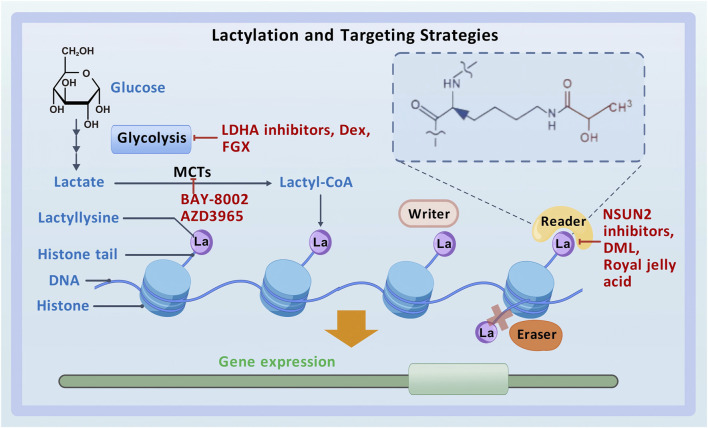
Lactylation and targeting strategies.

Lysine lactylation (Kla) is a novel type of PTM that directly stimulates gene transcription (Zhang et al., 2019) (Figure 1). Zhao et al. detected a 72.021-Da mass shift at the lysine residues of histones in human tumor cells using tandem mass spectrometry (MS/MS) in conjunction with high-performance liquid chromatography (HPLC). This mass shift was hypothesized to result from the addition of a lactyl group to lysine residues, which validated lactate as a substrate for histone modification and established histone lactylation as a PTM *in vivo* (Zhang et al., 2019; Huang et al., 2014). Lactyl-CoA donates a lactyl group to lysine residues on histone tails via p300 (Zeng et al., 2023). HBO1, a HAT belonging to the MYST family, has been identified as another lactyltransferase mediating histone lactylation-dependent gene transcription (Niu et al., 2024). In addition, HDAC1–3 have been identified as “erasers” for histone lactylation at histone H3 lysine 1 (H3K1) (Moreno-Yruela et al., 2022b). However, the enzymes that produce lactyl-CoA, the enzymes that derive the lactyl group, and other enzymes that participate in the regulation of histone lactylation remain unclear (Hou et al., 2024; Yang et al., 2023a), necessitating further investigation.

Studies have demonstrated that inhibition or promotion of glycolysis decreases or increases the total level of Kla, respectively, highlighting the sensitivity of histone lactylation to glycolysis-dependent lactate production (Jin et al., 2022; Irizarry-Caro et al., 2020). As an important component of the TME, lactate links metabolism, epigenetics, and transcription, thereby regulating gene expression. An immunosuppressive microenvironment is established and maintained in part by Kla. By directly binding to lactylation sites on the lysine residues of histones, lactate functions as an epigenetic regulator that directs the transcription of genes involved in damage repair, including arginase-1 (Arg1), and induces the polarization of macrophages toward the M2 phenotype through epigenetic reprogramming (Wang Z. H. et al., 2021). Pyruvate kinase M2 (PKM2) regulates glycolysis by directly interacting with lactate. In addition, lactylation at its K62 site enhances glycolytic activity, resulting in increased expression of Arg1. This regulation suppresses the inflammatory activity of M1 macrophages, promoting polarization toward the M2 phenotype (Wang et al., 2022a).

Histone lactylation exerts crucial epigenetic regulatory effects during tumorigenesis (Yu et al., 2021). Accumulation of lactate in the TME is a common feature of cancer that can induce histone lactylation, thus promoting the development of various types of tumors by disrupting the balance in gene transcription (Certo et al., 2021; Ippolito et al., 2019). Understanding the close relationship between histone lactylation and tumor development may offer novel avenues for developing more effective therapeutic strategies for cancer.

### 2.2 Non-histone lactylation

Lactylation can occur in non-histone proteins, affecting the structure and function of these proteins. Wan et al. found that peptides with lactylated lysine residues produced distinct immonium ions, which served as a marker for identifying lysine lactylation accurately. They discovered information regarding lactylation substrate proteins and modification sites by searching for these ions in existing human proteomic data resources. These findings suggest that lactylation plays an equally important regulatory role in non-histone proteins. Important cellular functions associated with both healthy and diseased states are mediated by these proteins (Wan et al., 2022). Identifying lactylation sites in non-histone proteins is another focus area of ongoing research in this field (Jing et al., 2024).

Lactylation can also occur in nuclear proteins. Studies have shown that the homologous recombination-associated protein MRE11 is lactylated at its K672 site via a p300-dependent mechanism. This modification leads to DNA end resection, promotes homologous recombination, and induces drug resistance in tumor cells (Chen et al., 2024b). Furthermore, the nuclear protein high-mobility group box-1 (HMGB1) can be lactylated (Yang K. et al., 2022) and subsequently secreted into the cytoplasm through exosomes. In the cytoplasm, lactylated HMGB1 regulates endothelial permeability, inflammation surrounding tumor cells, and stromal remodeling in the TME, thereby influencing the characteristics of the TME (Lan et al., 2020). Moreover, lactylation reduces the binding affinity of HMGB1 for immune cell surface receptors, impairing immune cell recognition and clearance of HMGB1, suppressing anti-tumor immune responses, and promoting cancer progression (Chen et al., 2024b). In a study, proteomic analysis of gastric cancer (GC) cells revealed HMGB1 among the 1,014 proteins with a total of 2,375 lactylation sites. The nucleus contained more than half of these lactylated proteins, whereas the remaining proteins were distributed across other cellular compartments. Lactylation is frequently found in spliceosomes and has been demonstrated to affect RNA splicing (Yang D. et al., 2022). Additionally, nuclear hypoxia-inducible factor 1-alpha (HIF1α) can undergo lactylation. In prostate cancer (PCa), the uptake of excess lactate via MCT1 results in lactylation of HIF1α, stabilizing the protein even under normoxic conditions. This stabilization activates downstream signaling, enhances transcriptional activity, and promotes angiogenesis (Luo et al., 2022). Alanyl-tRNA synthetase 1 (AARS1) has been identified as both a lactate sensor and a lactyltransferase that can lactylate p53 and promote tumorigenesis (Li H. et al., 2024; Ju et al., 2024). In a recent study, pan-cancer multi-omic analysis revealed overexpression of lactylation-related proteins such as glyoxalase 1 (GLO1), HDAC2, HDAC8, and HDAC1 in various tumor tissues (Wu Z. et al., 2024).

With advancements in scientific technology, more lactylation sites will be identified and their roles and regulatory mechanisms will be comprehensively investigated, offering new avenues for therapeutic research. However, studies on non-histone lactylation remain limited, necessitating further investigation into the functions and mechanisms of this modification.

## 3 Interactions between lactylation and metabolic pathways in tumors

### 3.1 Glycolysis

In normal cells, energy is derived primarily from OXPHOS or glycolysis. However, aerobic glycolysis or the Warburg effect is considered the predominant mechanism of energy production in tumor cells (Icard et al., 2018). Glycolysis produces high amounts of lactate, and subsequent histone lactylation can regulate gene expression. During glycolysis, glucose undergoes incomplete oxidation, generating two molecules of pyruvate. Pyruvate is reduced to lactate under the action of lactate dehydrogenase. Subsequently, acyltransferases catalyze the conversion of lactate to lactyl-CoA, which modifies lysine residues on histone tails, completing lactylation (Zhang et al., 2019). Therefore, a key element in histone lactylation is the imbalance between glycolysis and the tricarboxylic acid cycle (TAC). Although the glycolytic intermediate lactylglutathione (LGSH) is the source of the lactyl group in histone lactylation, high LGSH levels are associated with an increase in histone lactylation levels of glycolytic enzymes and a notable reduction in glycolysis. Consequently, histone lactylation acts as a feedback loop during glycolysis, with higher blood glucose levels promoting the formation of LGSH and lactylation, which in turn inhibits glycolysis (Gaffney et al., 2020).

Hypoxia is a hallmark of the microenvironment of solid tumors. Under hypoxic conditions, the upregulated expression of serine hydroxymethyltransferase 2 (SHMT2) enhances the glycolytic activity of esophageal cancer (EC) cells. Additionally, glycolysis-derived lactate induces lactylation of SHMT2, stabilizing the protein. Mechanistically, SHMT2 interacts with mitochondrial monofunctional C1-tetrahydrofolate synthase (MTHFD1L), promoting malignant progression in EC (Qiao et al., 2024). A recent study showed that a positive feedback loop involving glycolysis, H3K18 lactylation (H3K18la), and the protein kinases TTK and BUB1B exacerbates dysfunction in pancreatic ductal adenocarcinoma (PDAC) (Li et al., 2024b). These findings indicate that histone lactylation not only occurs during glycolysis but also influences glycolysis, playing a regulatory role in pathological processes (Wang M. et al., 2023). Understanding histone lactylation may provide novel insights into the mechanisms through which the Warburg effect contributes to cancer progression and introduce promising avenues for investigating the interplay between glycolysis and epigenetic modifications in cancer.

### 3.2 Acetylation

Recent studies have revealed complex crosstalk between histone lactylation and acetylation, both independently and synergistically, providing insights into their roles in tumorigenesis and their potential as therapeutic targets. Both lactylation and acetylation occur on lysine residues. p300, a member of the HAT family, has been identified as the primary “writer” enzyme for lactylation (Dai X. et al., 2022; Chen et al., 2021). Despite their similarities and shared catalytic enzymes, histone lactylation exhibits slower dynamic equilibrium kinetics (24 h) than histone acetylation (6 h), which suggests that acetylation occurs more readily than lactylation under physiological conditions (Dai X. et al., 2022). During lactylation, lactyl-CoA donates lactyl groups to lysine residues (Dai S. K. et al., 2022). Because both lactyl-CoA and acetyl-CoA are derived from pyruvate, the dominance of these pathways can vary across cell types (Gaur et al., 2021). Cells generate lactate under hypoxic conditions; however, the presence of sufficient oxygen promotes the conversion of pyruvate to acetyl-CoA, driving TAC (Zhang et al., 2019). The interaction between acetylation and lactylation has been shown to influence tumor progression. Deactivation of the mitochondrial pyruvate dehydrogenase complex (PDC) is necessary for cancer cells to transition from OXPHOS to aerobic glycolysis. According to a recent study, p300 acetylates the E3-binding protein (E3BP) of PDC at Lys 488, interfering with the interaction of PDHX with dihydrolipoyl transacetylase and preventing the assembly and activation of PDC. The majority of glucose is converted to lactate as a result of this disruption, which promotes aerobic glycolysis and H3K56 lactylation-mediated gene expression, eventually accelerating the growth of malignant tumors (Jiang Z. et al., 2024). A competitive link between histone lactylation and acetylation has also been observed, which indicates that several genes that cannot undergo acetylation are susceptible to lactylation. The ratio of lactylation to acetylation reflects the pathway of pyruvate conversion and may serve as an indicator of a cell’s susceptibility to malignancy (Dai X. et al., 2022). This competitive interplay highlights the importance of understanding the metabolic and epigenetic regulatory processes involved in tumorigenesis.

### 3.3 Other metabolic pathways

Lipid metabolism and the pentose phosphate pathway (PPP) have been associated with lactylation. Chen et al. conducted a comprehensive lactyl-proteomic and metabolomic analysis on samples from patients with non-small cell lung cancer (NSCLC). The findings revealed that intracellular lactate promotes extracellular lipolysis through lactylation of apolipoprotein C-II (APOC2). Mechanistically, lactate enhances lactylation of APOC2, stabilizing the protein and resulting in the release of free fatty acids, accumulation of Tregs, resistance to immunotherapy, and tumor metastasis (Chen J. et al., 2024). Another study demonstrated that lactate dehydrogenase A (LDHA) is activated under nutrient deprivation by UNC-51-like kinase 1 (ULK1), which phosphorylates serine-196 and promotes lactate production. Subsequently, lactylation of the autophagy-related protein vacuolar protein sorting 34 (Vps34) enhances its binding to phosphoinositide-3-kinase class 3 (PIK3C3) and increases its lipid kinase activity. This process, which facilitates autophagy and lysosomal degradation, is closely associated with cancer progression (Jia et al., 2023). Furthermore, a recent study on cervical cancer found that lactate induces lactylation of discoidin, CUB, and LCCL domain-containing protein 1 (DCBLD1) at the K172 site. This modification stabilizes DCBLD1 expression, leading to an increase in the expression and enzymatic activity of glucose-6-phosphate dehydrogenase (G6PD), which stimulates the PPP and promotes the progression of cervical cancer (Meng et al., 2024). Additionally, lactylation of NMNAT1 can support the survival of pancreatic cancer cells by maintaining the nuclear NAD + salvage pathway (Huang H. et al., 2024).

These findings highlight the intricate crosstalk between lactylation and other metabolic pathways. However, research in this area remains limited. Understanding this crosstalk and the underlying mechanisms may provide novel avenues for preventing tumor progression, developing targeted therapeutic approaches, and optimizing existing treatment strategies.

## 4 Role of lactylation in tumor progression

### 4.1 Role in tumor initiation and growth

Studies have shown a strong correlation between lactylation and tumor initiation. In colorectal cancer (CRC), the overall level of Kla is negatively correlated with the prognosis. Among the lactylated proteins identified in CRC, the lysine acetyltransferase KAT8 mediates the lactylation of elongation factor 1 alpha (eEF1A2) at the K408 site, enhancing elongation during translation and protein synthesis, thus promoting tumorigenesis (Xie et al., 2024). In a study on cutaneous melanoma (CM), Yu et al. found that histone lactylation facilitated tumorigenesis by promoting the expression of YTH N6-methyladenosine RNA-binding protein 2 (YTHDF2) (Yu et al., 2021). Similarly, in CRC, histone lactylation increases the stability of potassium channel subfamily K member 6 (Kcnk6) in a YTHDF2-dependent manner, enhancing the activity of potassium channels, inducing the activation of the NOD-like receptor thermal protein domain-associated protein 3 (Nlrp3) inflammasome, and eventually driving inflammation-induced carcinogenesis (Yuan et al., 2024).

Furthermore, lactylation is involved in tumor cell proliferation (Fu et al., 2024; Zang et al., 2024). Miao et al. demonstrated that hypoxia promoted lactylation of β-catenin, enhancing its stability and driving CRC cell proliferation via the Wnt signaling pathway (Miao et al., 2023). Insulin-like growth factor 1 (IGF1) and its receptor (IGF1R) play crucial roles in regulating glycolysis in tumors. In lung cancer, IGF1R is highly expressed and its lactylation is associated with the malignant proliferation of tumor cells. Lactate enhances the stability of IGF1R protein, forming a feedback loop that promotes glycolysis and lactate production (Zhang R. et al., 2024). The histone H3 variant CENPA interacts with yin and yang 1 (YY1) to promote the production of neuropilin 2 (NRP2) and cyclin D1 (CCND1). Lactylation of CENPA at K124 promotes its activation, increasing the expression of its target genes and accelerating tumor development in hepatocellular carcinoma (HCC) (Liao et al., 2023). The transcription of serine/arginine-rich splicing factor 10 (SRSF10) is enhanced by increased aerobic glycolysis, which also increases histone lactylation levels at the c-Myc promoter. This phenomenon promotes alternative splicing of BCL2-like 1 (Bcl-x) and murine double minute 4 (MDM4), facilitating the proliferation of HCC cells (Pandkar et al., 2023; Yao and Yang, 2024). In anaplastic thyroid cancer (ATC), aerobic glycolysis increases cellular lactate utilization and overall protein lactylation. Specifically, H4K12la activates genes essential for the proliferation of ATC cells. The oncogenic mutation BRAFV600E further promotes glycolysis, re-inducing lactylation and leading to H4K12la-driven dysregulation of transcription and the cell cycle (Wang X. et al., 2023). Li et al. performed immunohistochemical analysis on 60 triple-negative breast cancer samples and found increased levels of total lactylated proteins and specific histone H4K12 lactylation in the samples. H4K12la may bind to the schlafen 5 (SLFN5) promoter region, suppressing the expression of SLFN5 and leading to altered gene profiles and reduced apoptosis (Li J. et al., 2024).

Tumor cells exhibit remarkable plasticity (Chiodi and Mondello, 2020). The acquisition of stem cell-like properties is a major factor contributing to the self-renewal and long-term proliferation of cancer cells (Li Q. et al., 2024; Han et al., 2020). Lactate serves as a crucial metabolite, signaling molecule, and energy source during the development of HCC. Intracellular lactylation is considered a contributing factor in the development of HCC. Compared with HCC cells, liver cancer stem cells (LCSCs), the source of phenotypic and functional heterogeneity in HCC, exhibit enhanced glycolysis, lactate accumulation, and lactylation. H3K56la is closely associated with tumor initiation and the stemness of LCSCs. In a study, a comprehensive analysis of the lactylome and proteome of LCSCs showed that lactylation of aldolase A (ALDOA) at K230/322 enhanced the function of DDX17 in maintaining stemness. However, the impact of Kla on HCC stem cells remains unclear. Targeting lactylated ALDOA represents a promising therapeutic strategy for HCC (Feng et al., 2024). In glioblastoma (GBM), lactylation of histone H3 increases the expression of LINC01127, which directs polymerase II (POLR2A) to the mitogen-activated protein kinase kinase kinase kinase 4 (MAP4K4) promoter region. This interaction activates the JNK pathway, regulating the self-renewal of GBM cells (Li et al., 2023).

### 4.2 Role in tumor invasion and migration

Invasion and migration are hallmarks of malignant tumors and significantly impact treatment outcomes and prognosis. Approximately 80% of cases of renal cell carcinoma (RCC), one of the three main cancers of the urinary system, are attributed to clear cell renal cell carcinoma (ccRCC). According to a bioinformatic study, FK506-binding protein 10 (FKBP10) is essential for the glycolytic pathway that leads to metastasis in ccRCC. FKBP10 directly interacts with LDHA via its C-terminal region, enhancing the phosphorylation of LDHA-Y10. This process leads to an overactive Warburg effect and histone lactylation (Liu R. et al., 2024). Lactylation at H3K18 is associated with several malignant features in tumors (Hou et al., 2024). Zhou et al. found that G protein-coupled receptor 37 (GPR37) was significantly overexpressed in human CRC specimens and was correlated with a poor prognosis. Mechanistically, GPR37 promotes LDHA expression and glycolysis by activating the Hippo pathway, which increases H3K18la levels, upregulates chemokine CXC motif ligand 1 (CXCL1) and CXCL5, and enhances tumor cell migration (Zhou et al., 2023). In GC, dynamic H3K18 lactylation activates the transcription of vascular cell adhesion molecule 1 (VCAM1), which stimulates downstream AKT–mTOR signaling, thereby promoting the proliferation and migration of GC cells. Therefore, H3K18 lactylation may serve as a therapeutic target for GC (Zhao Y. et al., 2024). In HCC, proline-5-carboxylate reductase 1 (PYCR1) modulates the transcription of insulin receptor substrate 1 (IRS1) by influencing H3K18 lactylation in its promoter region, thus driving metastasis and promoting other malignant characteristics of HCC cells (Wang H. et al., 2024).

Epithelial–mesenchymal transition (EMT) is a crucial mechanism through which tumor cells acquire metastatic potential. EMT decreases cell-to-cell interactions and promotes migration and motility (Dongre and Weinberg, 2019). This process is hijacked in cancer to enable morphological and motility-related changes that stimulate invasion and migration (Lu et al., 2019). Glucose transporter 3 (GLUT3) plays an important role in glycolysis in cancer. In a study on GC, single-cell sequencing revealed significant upregulation of GLUT3 expression in primary and metastatic tumor tissues. This increased expression of GLUT3 was positively correlated with the expression of LDHA and the activity of lactylation-associated pathways and was functionally associated with EMT (Yang et al., 2023a). Lysyl oxidase (LOX) secreted by CAFs controls EMT through TGFβ/IGF1 signaling and enhances PD-L1 expression through histone lactylation (Li Z. et al., 2024). In pancreatic cancer, Rho GTPase Rif (RHOF) promotes the lactylation and nuclear translocation of Snail 1 by enhancing PKM2-mediated glycolysis, thereby inducing EMT (Zhao R. et al., 2024). In HCC, increased lactylation at H3K9 and H3K56 activates the transcription of endothelial-specific molecule 1 (ESM1) to promote EMT (Zhao P. et al., 2024).

Angiogenesis is crucial for tumor cells to acquire nutrients and metastasize (Zheng et al., 2023; Bhat et al., 2021). Studies have shown that the lactation of H3K9 in Endothelial cells can regulate Vascular Endothelial Growth Factor (VEGF), thereby inducing angiogenesis (Fan et al., 2024). PCa is one of the most prevalent cancers in adult men worldwide. Recent studies have shown that the expression of the hyaluronan-binding protein KIAA1199 is correlated with tumor stage, HIF-1α overexpression, and angiogenic markers in PCa. Overexpression of KIAA1199 enhances the production of secretory vascular endothelial growth factor A (VEGFA), promoting angiogenesis and vasculogenic mimicry (VM). In a study, ChIP-PCR and dual-luciferase reporter assay showed that HIF-1α enhances the transcription of KIAA1199. Mechanistically, lactate imported via MCT1 stabilizes HIF-1α through lactylation, enhancing the transcription of KIAA1199 (Luo et al., 2022). HCC is characterized by abundant vasculature. Upon its activation by histone lactylation and c-Myc, Golgi phosphoprotein 73 (GP73) leads to the phosphorylation of signal transducer and activator of transcription 3 (STAT3), which in turn enhances the pro-angiogenic function of GP73 (Ye et al., 2024).

These findings emphasize the crucial roles of lactylation in tumor invasion, migration, and angiogenesis, providing potential therapeutic targets for metastatic and vascularized tumors.

### 4.3 Role in immune regulation

Tumors form a complex ecosystem, with various immune cells being intricately involved. Studies have highlighted the important role of lactylation in immune regulation in tumors.

During tumor progression, cancer cells adopt diverse mechanisms to evade immune surveillance, such as downregulating antigen presentation pathways or inducing inhibitory immune checkpoints (Jhunjhunwal et al., 2021). Lactate-driven histone lactylation in KRAS-mutant tumor cells has been shown to activate the transcription of circATXN7, a circular RNA that interacts with NF-κB. circATXN7 sequesters p65 in the cytoplasm by binding to and masking the nuclear localization signal motif of the NF-κB p65 subunit, increasing the susceptibility of tumor-specific T cells to activation-induced cell death (AICD) and promoting immune evasion (Zhou et al., 2024). In NSCLC, H3K18la enhances immune evasion by activating the pore membrane protein 121 (POM121)/MYC/PD-L1 axis (Zhang C. et al., 2024). In GC, high lactylation levels are strongly correlated with poor overall survival and disease progression. Mechanistic experiments have shown that high lactylation levels are characterized by extensive macrophage infiltration, increased genomic instability, and a high risk of immune dysfunction in tumors (Yang et al., 2023b). In experimental models of PCa with PTEN/p53 abnormalities, activation of the Wnt/β-catenin pathway has been shown to result in lactylation at H3K18 in tumor cells and tumor-associated macrophages (TAMs), contributing to immune evasion (Chaudagar et al., 2023).

Cancer cells exploit immune cells, such as macrophages, CD8^+^ T cells and Tregs, to shape an immunosuppressive TME (Lei et al., 2020). Tumor-derived lactate is a critical driver of TAM polarization (Fang et al., 2023). For instance, Li et al. found that tumor-derived lactate promoted H3K18la, suppressing the transcription of RARγ in macrophages. This process enhanced IL-6 levels in the TME, activating STAT3 signaling in CRC cells and promoting the polarization of macrophages toward the M2 phenotype (Li X. M. et al., 2024). In neuroblastoma (NB), hexokinase-3 (HK3), a key enzyme in glucose metabolism, is correlated with M2 macrophage infiltration, increased lactate secretion by tumor cells, and histone lactylation (Wu X. et al., 2024). Proprotein convertase subtilisin/kexin type 9 (PCSK9) can inhibit M1 polarization by increasing lactate levels, promoting protein lactylation, and strengthening the activity of macrophage migration inhibitory factor (MIF) (Wang L. et al., 2022). In cervical cancer, lactate secreted by tumor cells increases H3K18la levels and promotes M2 polarization by upregulating glycerol-3-phosphate dehydrogenase 2 (GPD2), thereby supporting tumor progression (Huang C. et al., 2024). In advanced stages, monocyte-derived macrophages (MDMs) increase in number and lactate-driven histone lactylation increases IL-10 expression, forming an immunosuppressive TME by suppressing T-cell activity (De Leo et al., 2024).

Lactylation can influence the activity of T cells, one of the key components of the TME. Activation and functional differentiation of CD8^+^ T cells are regulated by lactate-associated metabolic pathways. Histone modifications such as H3K18la and H3K9la are enriched in CD8^+^ T cells, serving as transcriptional initiation factors for key regulatory genes (Raychaudhuri et al., 2024). SRSF10, which is upregulated in various tumors, enhances glycolysis-related enzymes, such as LDHA, increasing intracellular and extracellular lactate levels. This increase induces histone lactylation and amplifies SRSF10 expression. In addition, tumor-derived lactate can induce H3K18la in macrophages, activating transcription and enhancing cellular activity. M2 macrophages, in turn, inhibit CD8^+^ T cell infiltration, exacerbating the immunosuppressive TME (Cai et al., 2024).

**FIGURE 2:**
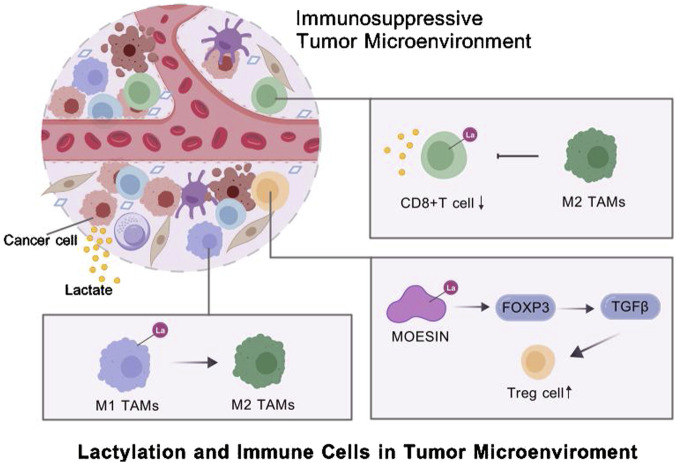
Lactylation and immune cells in tumor microenviroment.

Additionally, regulatory T cells (Tregs) are crucial for the development and maintenance of the suppressive TME (Li et al., 2020). A study showed that lactylation of lysine residues in membrane-organizing extension spike protein (MOESIN) increased the expression of forkhead box P3 (FOXP3) and enhanced the regulation of transforming growth factor-beta (TGF-β) signaling, thus sustaining Treg-mediated immunosuppression and promoting TME formation (Gu et al., 2022a). Lactylation at K72 in membrane proteins regulates Treg cell generation by enhancing their interactions with TGF-β receptors (Gu et al., 2022b). STAT5 expression promotes glycolysis in acute myeloid leukemia (AML) cells, leading to lactate accumulation. Excess lactate triggers the nuclear translocation and histone lactylation of E3BP, eventually inducing the transcription of PD-L1. Therefore, patients with AML characterized by STAT5-driven glycolysis and lactate accumulation may benefit from PD-1/PD-L1 blockade therapies (Huang et al., 2023) (Figure 2).

Tumor immune regulation is a critical aspect of understanding tumorigenesis, with profound implications for developing novel treatments, elucidating mechanisms underlying drug resistance, and improving patient prognosis. Lactylation has demonstrated substantial research potential in this domain. Ongoing studies will likely uncover novel therapeutic targets, advancing the development of novel immunotherapies.

### 4.4 Role in the genome

As a PTM, lactylation inevitably exerts significant effects on the genome. TIMs, crucial players in tumor immune evasion, are regulated by various epigenetic mechanisms. Lactylation-induced activation of the m6A–YTHDF1 axis mediated by methyltransferase-like 3 (METTL3) improves the translation efficiency of JAK1 and phosphorylation of STAT3, supporting the immunosuppressive properties of TIMs (Xiong et al., 2022). N1-methyladenosine (m1A) RNA modification serves as a key regulator of RNA metabolism. Histone lactylation can upregulate AlkB homolog 3 (ALKBH3), which in turn suppresses m1A modification of speckled protein 100A (SP100A), weakening the formation of tumor-suppressive promyelocytic leukemia (PML) protein condensates and facilitating the malignant transformation of tumor cells (Gu X. et al., 2024). Therapeutic resistance and tumor development are significantly influenced by cellular plasticity. In a study, multi-omic analysis showed that the expression of zinc finger E-box-binding homeobox 1 (ZEB1) is indicative of an intermediate state that tumor cells go through during the progression of neuroendocrine prostate cancer (NEPC). EMT, stemness, and neuroendocrine traits are present in this stage. ZEB1 directs tumor cells toward glycolysis for energy production by controlling the transcription of numerous important glycolytic enzymes. Histone lactylation induced by lactate accumulation during this process improves cellular plasticity and chromatin accessibility (Wang D. et al., 2024).

These findings highlight the essential role of lactylation in modulating genomic and epigenomic landscapes, influencing tumor cell behavior, and promoting malignancy. Understanding these mechanisms may offer promising avenues for targeted therapeutic interventions in cancer.

### 4.5 Role in treatment resistance

Despite significant advancements in the treatment of cancer, therapeutic resistance associated with molecular and clinical relapse remains prevalent, resulting in a poor prognosis. Therefore, understanding the mechanisms underlying therapeutic resistance is necessary to improve the treatment of cancer. Recent studies have shown that lactylation may contribute to treatment resistance in cancer (Takata et al., 2024).

Resistance to traditional anti-cancer treatments has been closely associated with lactylation in various tumor types. Lactylation of Nijmegen breakage syndrome protein 1 (NBS1) at lysine 388 (K388) is essential for the formation of the MRE11–RAD50–NBS1 (MRN) complex and the accumulation of DNA repair proteins at DNA double-strand break sites. However, this process is associated with adverse outcomes of neoadjuvant chemotherapy (Yang et al., 2023b). In metastatic CRC, bevacizumab is an important drug in first- and second-line treatments. Studies have shown that patients with bevacizumab-resistant CRC exhibit increased levels of histone lactylation, which promotes the transcription of recombinant rubicon-like autophagy enhancer (RUBCNL) through interactions with beclin 1 (BECN1). This process drives the maturation of autophagosomes, recruitment of the class III phosphoinositide 3-kinase complex, and proliferation and survival of cancer cells under hypoxic conditions (Li W. et al., 2024). Patients with bladder cancer (BCa) often develop resistance to cisplatin. H3K18la drives the transcription of Y-box-binding protein 1 (YBX1) and YY1, which are closely associated with cisplatin resistance (Li et al., 2024h). Similarly, histone lactylation plays a role in the development of cisplatin resistance in oral squamous cell carcinoma (OSCC) (Huang G. et al., 2024). In a study on GBM, interactions between aldehyde dehydrogenase 1 family, member A3 (ALDH1A3) and PKM2 were found to enhance PKM2 tetramerization in glioblastoma stem cells (GSCs), promoting lactate accumulation. Proteomic analysis of these GSCs revealed lactylation of X-ray repair cross-complementing protein 1 (XRCC1) at K247. Lactylated XRCC1 enhanced DNA repair, conferring drug resistance in ALDH1A3-overexpressing GBM cells (Li et al., 2024). Temozolomide (TMZ) resistance is a major barrier to the successful treatment of GBM. Studies have shown that H3K9la significantly enriches the LUC7L2 promoter, activating its transcription and promoting TMZ resistance in GBM (Yue et al., 2024). Other studies on the role of lactylation in the acquisition of resistance to standard anti-cancer therapies are highlighted in Table 1 (Lu Y. et al., 2024; Chu et al., 2023; Duan et al., 2023; Sun X. et al., 2023).

**TABLE 1:** Summary of lactylation’s functions in the malignant characteristics of cancers.

Malignant characteristics	Tumor type	Protein	Modification site	Mechanism	References
Tumorigenesis	Colorectal cancer	non-histone	K408 of eEF1A2	Enhance translation elongation and protein synthesis	Xie et al. (2024)
		histone	not clear	Increase the stability of Kcnk6 in a YTHDF2-dependent manner, inducing Nlrp3 activation	Yuan et al. (2024)
	Cutaneous melanoma	histone	H3K18	Promote the expression of YTHDF2	Yu et al. (2021)
Cell proliferation	Colorectal cancer	non-histone	β-catenin	Activate the Wnt signaling pathway	Miao et al. (2023)
	Lung cancer	non-histone	IGF1R	Form a feedback loop that promotes glycolysis and lactate production	Zhang et al. (2024a)
	Hepatocellular carcinoma	histone	K124 of histone H3 variant CENPA	Work with YY1 to promote the production of NRP2 and CCND1	Liao et al. (2023)
		histone	H3K18	Promote alternative splicing of Bcl-x and MDM4	Pandkar et al. (2023)
	Anaplastic thyroid cancer	histone	H4K12	Activate BRAFV600E, further promoting glycolysis, reconstructing the lactylation environment and leading to cell cycle dysregulation	Wang et al. (2024a)
	Triple-negative breast cancer	histone	H4K12	Suppress SLFN5 expression and lead to altered gene profiles and reduced apoptosis	Li et al. (2024c)
Generation of stem cell like characteristics	Hepatocellular carcinoma	non-histone	K230/322 of ALDOA	Enhance the regulatory function of DDX17	Feng et al. (2024)
	Glioblastoma	histone	H3K18	Increase the expression of LINC01127, which directs POLR2A to the MAP4K4 promoter region. This interaction activates the JNK pathway	Li et al. (2023)
Invasion and migration	Clear cell renal cell carcinoma	histone	Not clear	Not clear	Liu et al. (2024a)
	Colorectal cancer	histone	H3K18	Upregulate CXCL1 and CXCL5	Zhou et al. (2023)
	Gastric cancer	histone	H3K18	Activate the transcription of VCAM1, which subsequently stimulates downstream AKT-mTOR signaling	Zhao et al. (2024a)
	Hepatocellular carcinoma	histone	H3K18	PYCR1 modulates IRS1 transcription by influencing H3K18la in its promoter region	Wang et al. (2024a)
EMT	Gastric cancer	histone	H3K9, H3K18, H3K56, H4K8, H4K12	Not clear	Yang et al. (2023a)
		histone	H3K18	CAF-secreted LOX controls EMT via TGFβ/IGF1 signaling and enhances PD-L1 expression through histone lactylation	Li et al. (2024e)
	Pancreatic cancer	non-histone	Snail	RHOF enhances glycolysis mediated by PKM2, promoting lactylation and nuclear translocation of Snail 1, thereby inducing EMT	Zhao et al. (2024b)
	Hepatocellular carcinoma	histone	H3K9, H3K56	Activate the transcription of ESM1	Zhao et al. (2024c)
Tumor angiogenesis	Prostate cancer	non-histone	HIF-1α	Enhance KIAA1199 transcription, improving the production of secretory VEGFA	Luo et al. (2022)
	Hepatocellular carcinoma	histone	H3K18, H4K5	Activate GP73, causing STAT3 to become phosphorylated which further amplifies GP73-mediated pro-angiogenic functions	Ye et al. (2024)
Immune escape	Prostate cancer	histone	H3K18	Activate the transcription of circATXN7 sequestering p65 in the cytoplasm that make tumor-specific T cells more susceptible to AICD	Zhou et al. (2024)
	Non-small cell lung cancer	histone	H3K18	Activate the POM121/MYC/PD-L1 axis	Zhang et al. (2024b)
	Prostate cancer	histone	H3K18	Activation of the Wnt/β-catenin pathway results in H3K18la and TAMs with PTEN/p53 abnormalities	Chaudagar et al. (2023)
Immune suppression	Colorectal cancer	histone	H3K18	Suppress the transcription of RARγ in macrophages which enhances IL-6 levels in the TME, activating STAT3 signaling and fostering M2-like macrophage polarization	Li et al. (2024f)
		not clear	not clear	Inhibit M1 macrophage polarization	Wang et al. (2022b)
	Neuroblastoma	histone	not clear	M2 macrophage infiltration	Wu et al. (2024b)
	Cervical cancer	histone	H3K18	Promote M2 macrophage polarization	Huang et al. (2024b)
	Glioblastoma	histone	not clear	Boost IL-10 expression forming an immunosuppressive TME by suppressing T cell activity	De Leo et al. (2024)
	Hepatocellular carcinoma	histone	K72	Regulate Treg cell generation by enhancing interactions with TGF-β receptors	Gu et al. (2022b)
	Acute myeloid leukemia	histone	H3K18	Triggers E3BP nuclear translocation and induce PD-L1 transcription	Huang et al. (2023)
	Not clear	histone	H3K18, H3K9	Serve as transcriptional initiation factors for key regulatory genes	Raychaudhuri et al. (2024)
	Hepatocellular carcinoma	histone	H3K18	Amplify SRSF10 expression, activate transcription and boost TAM activity	Cai et al. (2024)
Impacts on genome	Colorectal cancer	non-histone	METTL3	Lactylation-induced activation of the m6A-YTHDF1 axis mediated by METTL3 improves JAK1 protein translation efficiency and STAT3 phosphorylation, supporting the immunosuppressive properties of TIMs	Xiong et al. (2022)
	Acute myeloid leukemia	histone	H3K18	Upregulate ALKBH3 expression, then reduce m1A methylation on SP100A weakening the formation of tumor-suppressive PML protein condensates and facilitating malignant cancer transformation	Gu et al. (2024a)
	Prostate cancer	histone	H3K18	Improve cellular plasticity and chromatin accessibility	Wang et al. (2024b)
Therapy resistance	Gastric cancer	non-histone	NBS1	Be essential for the formation of the MRN complex and the accumulation of DNA repair proteins	Yang et al. (2023b)
	Colorectal cancer	histone	H3K18	Promote the transcription of RUBCNL through interaction with BECN1 which drives autophagosome maturation, recruitment of the class III phosphoinositide 3-kinase complex, and cancer cell proliferation and survival under hypoxic conditions	Li et al. (2024g)
		histone	not clear	Activate the ALDOB/PDK1/lactate/CEACAM6 axis	Chu et al. (2023)
		histone	H4K12	Increase ABC transporter	Sun et al. (2023a)
	Bladder cancer	histone	H3K18	Drive the transcription of key transcription factors YBX1 and YY1	Li et al. (2024h)
	Oral squamous cell cancer	histone	H3K18, H3K27	Induce BCAM expression	Huang et al. (2024c)
	Glioblastoma	non-histone	XRCC1	Enhance DNA repair conferring therapy resistance in ALDH1A3-overexpressing	Li et al. (2024)
		histone	H3K9	Enrich the LUC7L2 promoter activating its transcription and promoting TMZ resistance	Yue et al. (2024)
	Hepatocellular carcinoma	non-histone	IGF2BP3	By maintaining high levels of PCK2 and NRF2, the lactylated IGF2BP3-PCK2-SAM-m6A loop strengthens the antioxidant system	Lu et al. (2024a)
	Lung cancer-derived brain metastasis	histone	H4K12	Activate the transcription of CCNB1 and accelerate the DNA replication and cell cycle	Duan et al. (2023)
	Lung squamous cell carcinoma	histone	not clear	Be linked to high SLC2A1 expression which has a negative correlation with TIGIT, CTLA4, LAG3, PD-1	Hao et al. (2024)
		non-histone	APOC 2	Resulte in FFA release	Chen et al. (2024c)
	Head and neck squamous cell cancer	histone	H3K9	Control downstream IL-11 through the JAK2/STAT3 pathway that activates immunological checkpoint genes in CD8^+^ T cells	Wang et al. (2024c)
Microorganism	Colorectal liver metastasis	non-histone	RIG-I	*E. coli* inhibits NF-κB recruitment to the Nlrp3 promoter in macrophages by inducing retinoic RIG-I lactylation, which reduces Nlrp3 transcription and mediates M2 macrophage polarization	Gu et al. (2024b)
	Colorectal cancer	histone	H4K8 H4K5 H3K18	Increase production of LINC00152	Wang et al. (2022c)
	HBV-related hepatocellular carcinoma	non-histone	9,256 sites	Lactylation-dependent metabolic adaption pathways	Y et al. (2023)
Cellular senescence	Lung cancer	histone	H4K8 H4K16	Control the production of TERT which lowers telomerase activity and causes telomere dysfunction, offering fresh information on the function of LKB1-mediated senescence	Liu et al. (2024b)
Autophagy	Cervical cancer	non-histone	TFEB	Enhance autophagic flux	Huang et al. (2024d)
	Colorectal cancer	histone	H3K18	Encourage autophagosome maturation, which is essential for hypoxic cancer cells to proliferate and survive	Li et al. (2024g)
	Lung cancer, Gastric cancer	non-histone	VPS34	Enhance the association of VPS34 with BECN1, ATG14, and UVRAG, promoting autophagy and lysosomal degradation pathways	Sun et al. (2023b)

Immune checkpoint blockade (ICB) therapies have shown remarkable efficacy across multiple tumor types. However, resistance to these therapies is increasingly being reported. The expression of solute carrier family two member 1 (SLC2A1), which encodes the GLUT1 protein, is different between lung squamous cell carcinoma (LUSC) tissues and normal lung tissues. In a study, GLUT1 was identified as an independent prognostic factor for LUSC. According to LASSO analysis, high protein lactylation levels were associated with high SLC2A1 expression. Furthermore, SLC2A1 expression was found to have a negative correlation with the expression of TIGIT, CTLA4, LAG3, PD-1, and other common immune checkpoints (Hao et al., 2024). Increased levels of histone lactylation have been associated with a poor response to immunotherapy in head and neck squamous cell cancer (HNSCC). H3K9la can control downstream interleukin-11 (IL-11), which activates the transcription of various immune checkpoint genes in CD8^+^ T cells through the JAK2/STAT3 pathway. In addition, H3K9la is strongly correlated with IL-11 expression and a poor response to immunotherapy in patients with HNSCC in clinical settings (Wang R. et al., 2024). APOC2 can also promote resistance to immunotherapy in NSCLC (Chen J. et al., 2024).

Understanding the mechanisms through which lactylation contributes to drug resistance is essential for improving treatment outcomes in patients with cancer.

### 4.6 Microorganisms participate in lactation modification

The microbiome may play an important role in tumor initiation, progression, and metastasis. Microorganisms can influence cancer-related processes by modulating immune responses, promoting inflammation, and producing carcinogenic substances. Numerous intratumoral bacteria, such as *E. coli*, have been found to reside within tumors and contribute to liver metastasis of colorectal cancer (CRLM). *E. coli* inhibits the recruitment of NF-κB to the Nlrp3 promoter in macrophages by inducing lactylation of retinoic acid-inducible gene 1 (RIG-I), which suppresses the transcription of Nlrp3 and mediates M2 polarization. The absence of Nlrp3 impacts the immunosuppressive activity of Tregs and the anti-tumor responses of CD8^+^ T cells (Gu J. et al., 2024). In a prospective study, Yang et al. investigated the lactylome of patients with HCC with hepatitis B virus (HBV) infection through integrated genomic and proteomic analysis of the tumor and surrounding liver tissues. The results revealed 9,275 lactylation sites; of which, 9,256 sites were found on non-histone proteins, indicating that Kla is a ubiquitous alteration that affects both histone and non-histone proteins and performs other functions in addition to transcriptional regulation. Furthermore, analysis of the lactylome revealed lactylation-dependent metabolic adaption pathways in HBV-related HCC (Y et al., 2023). Gut microorganisms can play a role in disease development by interacting with the host genome through epigenetic factors, such as long non-coding RNAs (lncRNAs). Wang et al. measured transcriptomic alterations in a human colon cell line infected with the common gut pathogen *Salmonella typhimurium* SL1344. The results showed that bacterial lipopolysaccharides (LPSs) led to increased production of LINC00152, which was controlled by histone lactylation. The expression of LINC00152 was higher in CRC tissues than in adjacent normal tissues (Wang et al., 2022c).

### 4.7 Other mechanisms

In addition to its well-known tumor-suppressive function in lung cancer, liver kinase B1 (LKB1) is reported to be involved in cellular senescence. In LKB1-deficient A549 cells, overexpression of LKB1 increases H4K8 and H4K16 levels and causes lactate generation. By controlling the production of telomerase reverse transcriptase (TERT), this inhibition reduces telomerase activity and causes telomere dysfunction, providing novel insights into the function of LKB1-mediated cellular senescence in lung cancer (Liu M. et al., 2024).

**FIGURE 3:**
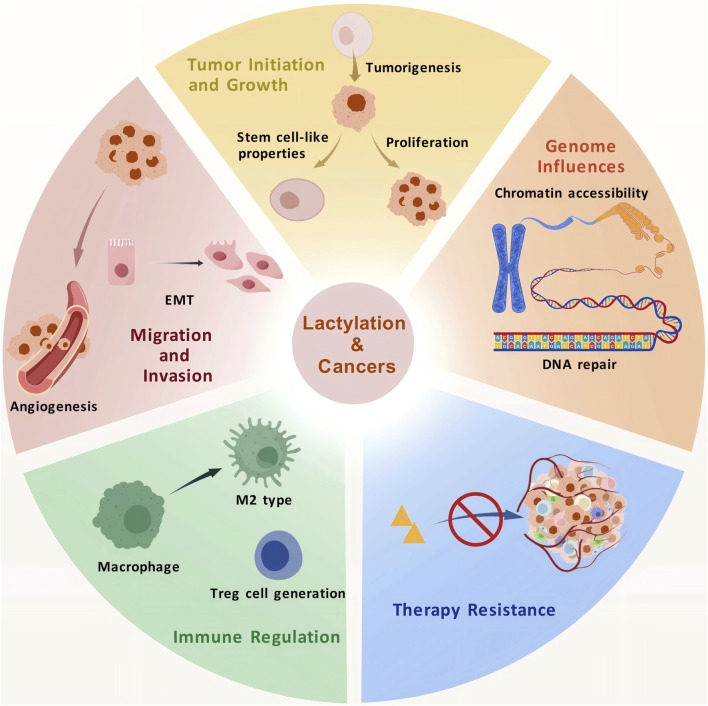
The roles of lactylation in tumor progression.

Autophagy is a lysosome-mediated self-degradation process that recycles damaged organelles and long-lived proteins, maintaining cellular homeostasis under metabolic stresses, such as starvation and hypoxia. Lactate has been shown to regulate autophagy. Transcription factor EB (TFEB), a key regulator of lysosomal biogenesis and autophagy, undergoes covalent lactylation, leading to its stabilization and increased activity, which enhances autophagic flux (Huang Y. et al., 2024). Furthermore, histone lactylation promotes autophagosome maturation, which is essential for the proliferation and survival of hypoxic cancer cells (Li W. et al., 2024). Lactylation of PIK3C3/VPS34 at K356 and K781 through lysine acetyltransferase 5 (KAT5) can enhance the association of VPS34 with BECN1, ATG14, and UVRAG, promoting autophagy and other lysosomal degradation pathways (Sun W. et al., 2023) (Figure 3).

## 5 Predicting clinical prognosis using lactylation-related genes

With the emergence of new bioinformatic technologies, such as machine learning, and publicly available databases, clinicians can analyze gene expression profiles to predict patient prognosis in cancer (Yin et al., 2024; Zheng et al., 2024; Pan et al., 2024; Cai et al., 2023). Studies have shown that lactylation is closely associated with the clinical outcomes of patients with cancer (Chen et al., 2024d; Lu X. et al., 2024; Liu J. et al., 2024). Feng et al. used machine learning algorithms to construct a risk model for CM based on lactylation-related genes using data from the TCGA and GEO databases. Eight genes, namely, TMSB4X, CRABP2, WAS, LAP3, SATB1, GATAD2A, CALML5, and MDC1 were identified as candidate lactylation-related genes for constructing the model. Patients in the TCGA-CM cohort were divided into high- and low-risk groups based on the median risk score. In the high-risk group, the expression levels of CRABP2, GATAD2A, and MDC1 were substantially high, whereas those of LAP3, SATB1, and WAS were low. In both TCGA and GEO cohorts, survival analysis revealed a negative correlation between the risk scores and overall survival (OS) in patients with CM (Feng et al., 2023). In another study, machine learning revealed SMAD4, KRAS, TTN, CDKN2A, and TP53 as the top five mutated genes in PDAC. In addition, the researchers investigated mutation trends in different PDAC subgroups based on lactylation scores. The tumor mutation burden (TMB) was positively correlated with lactylation scores, with high-scoring clusters having a greater TMB than low-scoring clusters. Compared with patients with low TMB and lactylation scores, those with high TMB and lactylation scores had notably shorter OS (Peng et al., 2024). In a study on cervical cancer, lactylation-related genes (KRGs) were used to classify tumors into two subtypes, C1 and C2. The C2 cluster exhibited suppressed glycolysis and enhanced OXPHOS, which corresponded to higher survival rates. Additionally, a prognostic model based on two lactate signaling genes, ISY1 and PPP1R14B, was developed. A negative correlation was observed between PPP1R14B expression and CD8^+^ T cell infiltration, which was associated with low survival rates. The prognostic model was validated in two independent cohorts of patients with cervical cancer, one treated in Uganda (n = 106) and the other in Seoul (n = 300). Survival analysis revealed considerably lower OS or disease-free survival (DFS) rates among high-risk patients (He et al., 2024). Chao et al. investigated the relationship between lactylation and OS or progression-free survival (PFS) in ovarian cancer. Higher H3K18la levels were associated with worse PFS (p < 0.001) and OS (p = 0.028). Multivariate Cox regression analysis showed that H3K18la was an independent risk factor for PFS (p = 0.001). Moreover, H3K18la levels were correlated with the tumor stage (p = 0.037). Altogether, high H3K18la levels were associated with a poor prognosis in epithelial ovarian cancer (Chao et al., 2024).

Understanding the role of lactylation in cancer prognosis can assist clinicians in devising personalized treatment plans, evaluating therapeutic efficacy, and monitoring adverse reactions (Hao et al., 2024; Yu et al., 2024). As mentioned earlier, lactylation is found in various tumor types; however, studies investigating its clinical prognostic value remain limited. Future research in this area is essential to broaden the understanding and application of lactylation-related biomarkers in cancer.

## 6 Therapeutic strategies targeting lactylation

Lactylation plays an important role in the development and progression of various tumors, making it a promising therapeutic target for cancer (Li X. M. et al., 2024; Sun L. et al., 2023). With the deepening of research on lactylation in recent years, numerous targeted therapies have been developed for cancer, primarily encompassing the following three categories: inhibitors of lactate metabolism, lactate transporter proteins, and lactylation sites (Figure 1).

LDHA inhibitors, dexmedetomidine (Dex), and fargesin (FGS) are inhibitors of lactate metabolism. In patients with HCC, the combination of anti-PD-1 therapy and LDHA inhibitors has demonstrated stronger anti-tumor effects than monotherapy with anti-PD-1 antibodies (Gu et al., 2022b). In GBM, the LDHA inhibitor oxamate can enhance the efficacy of CAR-T cell therapy by inhibiting the lactylation of exonucleases and C-C motif chemokine receptor 8 (CCR8) (Sun T. et al., 2023). In addition, Dex can suppress glycolysis and reduce lactylated c-Myc levels, thereby decreasing the stability of c-Myc protein and inhibiting the migratory and invasive abilities and glycolytic activity of GBM cells (Zhu and Zhang, 2024). In NSCLC, FGS interacts with PKM2 to regulate glycolysis and pyruvate metabolism, suppressing lactate production and LDHA expression. Moreover, it inhibits tumor-associated histone lactylation, thereby exerting anti-tumor effects (Guo et al., 2024).

As mentioned earlier, lactate is transported between cells via MCTs. MCT4, located on glycolytic tumor cells, transports the lactate produced by these cells to the intercellular space. This lactate is subsequently internalized by oxidative tumor cells via MCT1. BAY-8002 and AZD3965 are two potent inhibitors of MCT1 that can alter its open conformation and inhibit lactate transport in the TME (Wang N. et al., 2021).

Inhibitors of lactylation sites directly target lactylation to achieve anti-cancer effects. Accumulation of lactate in CRC cells activates the transcription of the m5C methyltransferase NSUN2 via H3K18la. Lactylation of NSUN2 at K356 contributes to its oncogenic role. Therefore, combining NSUN2 inhibitors with immunotherapy represents a promising therapeutic strategy for CRC (Chen B. et al., 2024). Cancer stem cells can reduce the effectiveness of anti-cancer medications by driving tumor development, progression, and recurrence. A triterpenoid anti-cancer substance called dihydrocelastrol (DML) prevents carcinogenesis in liver cancer by interfering with histone lactylation induced by metabolic stress (Pan et al., 2022). Royal jelly acid, a component of traditional Chinese medicine, exerts inhibitory effects on lactylation. For instance, it suppresses H3K9la and H3K14la to reduce tumorigenicity in HCC (Xu et al., 2023).

Effective therapeutic strategies targeting lactylation are lacking at present. Therefore, developing broad-spectrum inhibitors of lactylation is necessary. Lactylation plays an oncogenic role in various tumor types, thus serving as a promising therapeutic target for cancer. Further studies are warranted to explore and optimize lactylation-targeted strategies for the treatment of cancer in clinical settings.

## 7 Discussion

While lactylation research is still in its early stages, emerging evidence highlights lactylation’s potential as both a diagnostic/prognostic biomarker and a therapeutic target across diverse cancer types. With continued investigation into its regulatory enzymes, context-specific roles, and clinical translation, lactylation-based strategies may pave the way for more precise, metabolism-driven cancer therapies that can enhance patient outcomes and overcome resistance mechanisms.

Despite the growing interest in targeting lactylation as a cancer therapeutic strategy, several critical knowledge gaps and shortcomings hinder its clinical translation. First, the enzymatic machinery that governs histone lactylation remains incompletely characterized. Although p300 and HBO1 have been identified as potential lactyltransferases, the full complement of “writers,” “erasers,” and “readers” of lactylation is largely unknown, including the enzymes responsible for generating and regulating lactyl-CoA. This limited understanding impairs our ability to design specific modulators of lactylation. Second, most of the compounds described—such as LDHA inhibitors, oxamate, and dexmedetomidine—impact broad metabolic or epigenetic pathways rather than selectively targeting lactylation, raising concerns about off-target effects and therapeutic specificity.

Another significant gap lies in the tumor-type specificity and context dependency of lactylation. While lactylation appears to promote tumor progression in various cancers, it is not yet clear why certain tumor types rely more heavily on lactylation or how tumor microenvironmental differences shape lactylation dynamics. This lack of precision complicates the development of broadly effective therapies. Moreover, there is a noticeable absence of clinically validated biomarkers or non-invasive tools to measure lactylation activity in patients, making it difficult to stratify patients or monitor therapeutic responses.

Importantly, although some studies suggest lactylation plays a role in modulating the immune microenvironment—such as through macrophage polarization and Treg activation—detailed mechanistic insights into how lactylation-targeted therapies influence immune cell function are missing. Lastly, while initial combinations with immunotherapies have shown promise, systematic evaluations of combination strategies and potential resistance mechanisms to lactylation-targeted agents are lacking. Addressing these gaps through mechanistic, translational, and clinical research will be essential for realizing the full therapeutic potential of lactylation in cancer.

## 8 Conclusion

Lactylation, a PTM first identified in 2019, has received increased attention from researchers over the past 5 years for its important role in cancer. It not only participates in tumor pathogenesis but also serves as a potential prognostic biomarker and therapeutic target. Studies have demonstrated that lactylation impacts key malignant features, such as tumor initiation, growth, metastasis, immune regulation, and therapeutic resistance. Furthermore, genes associated with lactylation are closely related to clinical outcomes, highlighting the potential of lactylation as a therapeutic target. However, using lactylation as a diagnostic biomarker or therapeutic target is associated with several challenges. As discussed earlier, lactylation sites associated biological processes, and the regulatory molecules or pathways involved in lactylation vary across tumor types, complicating the development of lactylation as a universal therapeutic target for cancer. Moreover, due to the lack of universal and efficient detection methods, research on the prediction of patient clinical prognosis related to lactylation-modified genes is still insufficient in many tumor types.In addition, effective and selective lactylation inhibitors are still lacking. During the development of new inhibitors, the possibility of off-target effects, the challenges of tissue-specific drug delivery, and the need for reliable biomarkers to monitor treatment responses should also be taken into account. Despite these challenges, lactylation holds promise as a highly effective diagnostic/prognostic biomarker and therapeutic target for cancer owing to its unique role in disease progression.

Existing studies have introduced valuable avenues for investigating the pathophysiological, diagnostic, prognostic, and therapeutic roles of lactylation in tumors. Future research directions may focus on high-throughput screening to identify lactylation regulators, *in vivo* studies to test therapeutic effects, development of tissue-specific delivery systems, or the use of advanced techniques such as ChIP-seq or single-cell RNA-seq to describe lactylation dynamics in the tumor microenvironment. Further in-depth and innovative research will improve the understanding of the role of lactylation in tumor progression, laying a robust theoretical foundation for the clinical application of lactylation-targeted strategies. This progress may significantly benefit patients that alleviating the disease burdens caused by malignant tumors.

## Glossary

TME: tumor microenvironment
PTM: post-translational modification
OXPHOS: oxidative phosphorylation
MCTs: monocarboxylate transporters
CAFs: cancer-associated fibroblasts
HAT: histone acetyltransferase
HDACs: histone deacetylases
Kla: lysine lactylation
MS/MS: mass spectrometry
HPLC: high-performance liquid chromatography
H3K1: histone H3 lysine 1
Arg1: arginase-1
PKM2Pyruvate kinase M2TIMs: Pyruvate kinase M2TIMstumor-infiltrating myeloid cells
Treg: regulatory T cells
MOESIN: membrane-organizing extension spike protein
TGF-β: transforming growth factor-β
HMGB1: high mobility group box-1
GC: gastric cancer
HIF1α: hypoxia-inducible factor 1α
PCa: prostate cancer
AARS1: Alanyl-tRNA synthetase 1
GLO1: glyoxalase 1
TAC: tricarboxylic acid cycle
LGSH: lactylglutathione
SHMT2: serine hydroxymethyltransferase 2
EC: esophageal cancer
MTHFD1L: mitochondrial monofunctional C1-tetrahydrofolate synthase
H3K18la: H3K18 lactylation
PDAC: pancreatic ductal adenocarcinoma
E3BP: E3 binding protein
PDC: dehydrogenase complex
PPP: pentose phosphate pathway
NSCLC: non-small cell lung cancer
APOC2: apolipoprotein C-II
LDHA: lactate dehydrogenase A
ULK1: UNC-51-like kinase 1
PIK3C3: phosphoinositide-3-kinase Class 3
Vps34: vacuolar protein sorting 34
DCBLD1: Discoidin
CUB: and LCCL domain-containing protein 1
G6PD: glucose-6-phosphate dehydrogenase
CRC: colorectal cancer
eEF1A2: Elongation Factor 1 Alpha
CM: cutaneous melanoma
YTHDF2: YTH N6-methyladenosine RNA-binding protein 2
Kcnk6: Potassium Channel Subfamily K Member 6
Nlrp3: NOD-like receptor thermal protein domain-associated protein 3
IGF1: Insulin-like growth factor 1
YY1: Yin and Yang 1 protein
CCND1: Cyclin D1
NRP2: Neuropilin 2
HCC: hepatocellular carcinoma
SRSF10: Serine/Arginine-Rich Splicing Factor 10
Bcl-x: BCL2-like 1
MDM4: Murine Double Minute 4
ATC: anaplastic thyroid cancer
SLFN5: Schlafen 5
LCSCs: Liver cancer stem cells
ALDOA: Aldolase A
GBM: glioblastoma
POLR2A: Polymerase II
MAP4K4: Mitogen-Activated Protein Kinase Kinase Kinase Kinase 4
RCC: Renal cell carcinoma
ccRCC: clear cell renal cell carcinoma
FKBP10: FK506 Binding Protein 10
GPR37: G Protein Coupled Receptor 37
CXCL1: chemokine CXC motif ligand 1
VCAM1: Vascular Cell Adhesion Molecule 1
PYCR1: Proline-5-Carboxylate Reductase 1
IRS1: Insulin Receptor Substrate 1
EMTEpithelial-mesenchymal transitionGLUT3: Epithelial-mesenchymal transitionGLUT3Glucose Transporter 3
LOX: lysyl oxidase;
RHOF: Rho GTPase Rif
ESM1: Endothelial-Specific Molecule 1
VEGFA: Vascular Endothelial Growth Factor A
VM: vasculogenic mimicry
GP73: Golgi Phosphoprotein 73
STAT3: Signal Transducer and Activator of Transcription 3
AICD: activation-induced cell death
POM121Pore Membrane Protein 121TAM: Pore Membrane Protein 121TAMstumor-associated macrophages
NB: neuroblastoma
HK3: Hexokinase-3
PCSK9: Proprotein Convertase Subtilisin/Kexin Type 9
MIF: macrophage migration inhibitory factor
GPD2: Glycerol-3-Phosphate Dehydrogenase 2
MDMs: monocyte-derived macrophages
AML: acute myeloid leukemia
METTL3: methyltransferase-like 3
ALKBH3: AlkB homolog 3
SP100A: Speckled Protein 100A
PML: promyelocytic leukemia
NEPC: neuroendocrine prostate cancer;
ZEB1: Zinc Finger E-Box Binding Homeobox 1
m1A: N1-methyladenosine
NBS1: Nijmegen Breakage Syndrome Protein 1
K388: lysine 388
MRN: MRE11-RAD50-NBS1
BECN1: Beclin 1
BCa: Bladder cancer
RUBCNL: Recombinant Rubicon-Like Autophagy Enhancer Protein
YBX1: Y-box binding protein 1
OSCC: oral squamous cell carcinoma
ALDH1A3: Aldehyde Dehydrogenase 1 Family, Member A3
GSCs: glioblastoma stem cells
XRCC1: X-ray Repair Cross-Complementing Protein 1
TMZ: Temozolomide
ICB: Immune checkpoint blockade
LUSC: lung squamous cell carcinoma
SLC2A1: solute carrier family 2 member 1
HNSCC: head and neck squamous cell carcinoma
IL-11: interleukin-11
CRLM: colorectal liver metastasis
RIG-I: retinoic acid-inducible gene 1
HBV: hepatitis B virus
lncRNAslong non-coding RNAsLPS: long non-coding RNAsLPSlipopolysaccharides
LKB1: liver kinase B1
TERT: telomerase reverse transcriptase
TFEB: Transcription factor EB
KAT5: Lysine Acetyltransferase 5
OS: overall survival
TMB: Tumor mutation burden
KRGs: lactylation-related genes
DFS: disease-free survival
PFS: progression-free survival
CCR8: C-C Motif Chemokine Receptor 8
Dex: dexmedetomidine
K356: lysine 356
DML: Dihydrocelastrol
FGS: Folylpolyglutamate synthase
